# Access to and use of improved water and sanitation facilities in Sub-Saharan Africa: Analysis of Demographic and Health Survey Data from 30 countries

**DOI:** 10.1371/journal.pgph.0006195

**Published:** 2026-10-06

**Authors:** Joyce Aputere Ndago, Paul Adjorlolo, Martin Nyaaba Adokiya, Micheal Boah

**Affiliations:** 1 Department of Social and Behavioral Sciences, School of Public Health, University of Ghana, Accra, Ghana; 2 Department of Social and Behavioral Change, School of Public Health, University for Development Studies, Tamale, Ghana; 3 Department of Biostatistics, School of Public Health, College of Health Sciences, University of Ghana, Accra, Ghana; 4 Department of Global Health, School of Public Health, University for Development Studies, Tamale, Ghana; 5 Centre for Population Health, University of Global Health Equity, Kigali, Rwanda; African Population and Health Research Center, KENYA

## Abstract

Access to and use of improved water sources and sanitation facilities in Sub-Saharan Africa (SSA) remains a significant public health concern, with notable variations across countries. Achieving the Sustainable Development Goals related to water and sanitation require evidence on current levels of access and factors that shape them. This study examined household access to and use of improved water sources and sanitation facilities, as well as the determinants of their use in SSA. Demographic and Health Survey data from 465,208 households across 30 SSA countries conducted between 2014 and 2025 was used for this study. Descriptive analyses examined access levels. Multivariable Poisson regression with robust variance was used to identify the factors associated with reported use. Overall, 76.3% of households had access to and use of improved water sources, 56.7% had access to improved toilets, and 35.4% had access to basic sanitation facilities (unshared improved toilet facilities), with wide variations across countries. Significant factors associated with access and use of improved toilet and sanitation facilities included wealth and residence, while educational attainment was significantly associated with access to and use of improved water sources. The findings highlight that disparities in access to water and sanitation exist in the SSA region, with household wealth, place of residence, and education significantly associated with households’ access and use of improved water sources and sanitation facilities. Governments in SSA should prioritize improving access to water and sanitation, particularly in underserved areas. Additionally, targeted strategies are needed to address disparities driven by socioeconomic and demographic factors.

## Introduction

Access to and use of improved water sources and sanitation facilities play a critical role in public health and disease prevention, and for human survival [1]. The United Nations (UN) in 2010, through Resolution 64/292, affirmed access to clean water and basic sanitation facilities for all as a human right [1–3]. Approximately 1.2 billion people worldwide lack access to safe drinking water, and around 2.5 billion do not have adequate sanitation [2–4]. Inadequate access to safe water and good sanitation leads to an estimated annual death of 1.4 million due to inadequate access to safe water and good sanitation facilities [3,5]. Achieving access to safe water and sanitation continues to present significant challenges across various socio-demographics in sub-Saharan African (SSA) countries, despite the progress achieved in the last decade through Water, Hygiene and Sanitation (WASH) interventions [6,7].

Efforts to improve access to and use of improved water and sanitation facilities are interconnected, as both are vital components of sustainable development. Improvement in access to safe and improved water sources and sanitation may lead to better health outcomes for communities, enhance livelihoods, and improve overall quality of life. Consequently, safe WASH services are fundamental to achieving sustainable development as they constitute a critical prerequisite for health and wellbeing for every population [2,8,9]. Sustainable Development Goal (SDG) target 6.1 emphasizes the need for universal and equitable access to safe and affordable drinking water. Likewise, target 6.2 aims to ensure access to improved sanitation facilities which are equitable and adequate for all, with a specific focus on eliminating open defecation. This target is monitored using indicator 6.1.1, which refers to “safely managed drinking water services” [2]. This indicator is defined as drinking water from an improved water source that is situated on-premises, available when needed, and free from faecal and priority chemical contamination [2,3]. Additionally, Halkos and Tzeremes reported that the economic efficiency of SSA countries is positively impacted by population access to improved water sources and sanitation facilities [10].

Despite the significant improvement in access to water for nearly every region, substantial gaps in access persist [11,12]. According to the WHO, water demand in some developing countries is projected to exceed supply by 50% no later than 2030 [4,5]. Similarly, it is reported that the world remains off track to achieving universal coverage of access to and safe management of WASH services for many populations [7]

Polluted water and inadequate sanitation can significantly heighten the risk of various diseases, including cholera, typhoid, schistosomiasis, and certain cancers due to exposure to carcinogens [6,13,14]. Though access to safe water is crucial for promoting hygienic practices and preventing diseases and infection, research on access to and use of improved sources is limited [6,12,15]. Additionally, evidence on regional distribution and the factors that contribute to access and use remain limited. This study aimed to determine household access to and use of improved water sources and sanitation facilities in SSA.

## Materials and methods

### Ethics statement

The DHS survey incorporates the collection of biomarkers and is subjected to review and approval by the ethics review committee of the implementing country and the institutional Review Board of ICF International.

The original DHS survey protocols and instruments were reviewed and approved by the Inner-City Fund (ICF) Institutional Review Board (IRB) and complies with the U.S. Department of Health and Human Services regulations for the protection of human subjects (45 CFR 46). In addition, each participating country obtained approval from its respective national ethics or institutional review board, and all survey participants provided informed consent. Data used for this study is available at https://dhsprogram.com/data/available-datasets.cfm. The current study did not require ethical approval as it was a secondary analysis of the de-identified dataset. However, permission was obtained from the DHS program through ICF International to use the selected datasets.

### Data source and sample

This study used data from the most recent Demographic and Health Surveys (DHS) conducted in SSA from 2014 to 2025. The DHS data provides information on households’ access to water sources, toilet and sanitation facilities. They also provide information on wealth index, type of place of residence, sex of household head, and educational level. The DHS questionnaire is standardized and pre-tested to ensure compatibility across populations over time. Water and sanitation data is collected at both the individual and household levels in the DHS.

The DHS sampling frame was provided by the different country-level Population and Housing Censuses. The sampling design uses a two-stage stratified cluster sample methodology. Thus, the sample obtained is nationally representative. In the first stage, probability proportion-to-size was used to select Enumeration Areas (EAs) or clusters. Before the selection of EAs, they are stratified by region and then into urban and rural areas. In the second stage, a representative sample of households is drawn. The details of the DHS methodology are explained elsewhere in the guide to DHS Statistics, DHS 8 [16].

The current analysis incorporated data from 30 countries. Table 1 provides details on survey years and each country’s contribution to the overall sample. Missing data were evaluated for all study variables prior to analysis. The proportion of missing observations exceeded 5% for several primary outcomes and key covariates, indicating a non‑trivial level of missingness that required careful methodological consideration. To minimize bias arising from model‑dependent assumptions, we opted against imputing the primary outcome variables, as imputing binary outcome measures can introduce artificial precision and distort estimates in pooled, multi‑country datasets. Instead, we employed a complete‑case analysis for all multivariable models, whereby households with missing data on any outcome or covariate were excluded from the analytic sample. This approach ensured that estimated associations were based solely on observed values, avoiding the risk of biased or unstable estimates that may occur when imputing outcomes with heterogeneous country‑level patterns of missingness. To address potential concerns regarding representativeness, survey weights were retained to adjust for household non‑response, aligning the analysis with the underlying sampling design.

**Table 1:** Countries with weighted samples and survey years.

Country	Survey year	Weighted N	Weighted %
Angola	2015-2016	14,379	3.1
Benin	2017-2018	15,928	3.4
Burkina Faso	2021	17,659	3.8
Burundi	2016-2017	17,269	3.7
Cameroon	2018-2019	14,677	3.2
Chad	2014-2015	17,719	3.8
Cote d’Ivoire	2021	14,877	3.2
DR Congo	2014	4,181	0.9
Gabon	2019-2021	11,043	2.4
Gambia	2019-2020	11,865	2.6
Ghana	2022-2023	15,014	3.2
Guinea	2018	10,874	2.3
Kenya	2022	32,156	6.9
Lesotho	2023-2024	6,413	1.4
Liberia	2019-2020	8,065	1.7
Madagascar	2021	18,869	4.1
Malawi	2015-2016	24,562	5.3
Mali	2018	10,519	2.3
Mauritania	2019-2021	15,714	3.4
Mozambique	2022-2023	13,183	2.8
Nigeria	2018	41,821	9.0
Rwanda	2019-2020	14,634	3.2
Senegal	2023	16,583	3.6
Sierra Leone	2019	15,574	3.4
South Africa	2016	8,514	1.8
Tanzania	2022	15,254	3.3
Togo	2014	5,763	1.2
Uganda	2016	18,506	4.0
Zambia	2018-2019	13,683	2.9
Zimbabwe	2015	19,910	4.3
Total	2015-2016	465,208	100.0

After thoroughly cleaning the data to remove invalid and missing entries, 454,545 households were analyzed for water, and 454,480 for toilet and basic sanitation. To the best of the authors’ knowledge, they did not find specific pooled analysis studies published anywhere.

### Variables

#### Dependent variables.

The dependent variables in this study are “access to improved water sources,” “access to improved toilet facilities,” and “access to basic sanitation.” The WHO/UNICEF Joint Monitoring Programme (JMP) report reviewed the definition of improved water sources and sanitation with additional criteria relating to service levels [17]. According to JMP, improved water sources include piped water into a dwelling, piped water to a yard or plot, public standpipes, boreholes or tubewells, protected dug wells, protected springs, rainwater collection, and packaged or delivered water (if from an improved source and used for cooking and hygiene). Improved toilet facilities include flush or pour flush toilets connected to a piped sewer system, septic tank, or pit latrine, ventilated improved pit (VIP) latrines, pit latrines with slabs, and composting toilets. Households that reported using any of the above water or toilet facilities were coded as ‘1’ to indicate access to an improved facility while those that did not report using improved facilities were coded as ‘0.’ Access to basic sanitation was defined as having access to improved toilet facilities that were not shared with other households.

#### Independent variables.

The independent variables considered for this study were chosen based on their availability in the DHS data, the objectives of the study, and the literature reviewed. The variables consisted of households’ place of residence (urban/rural); sex of the household head (male or female); educational level and wealth index (poorer, poor, middle, richer and richest). The wealth quintile was pre-defined in the data set using various variables relating to household characteristics and amenities, based on the principal component analysis. All variables were used as they existed in the dataset after cleaning.

### Data analysis

Data analysis was conducted using STATA 17.0 and involved descriptive, bivariate, and multivariable modelling approaches. Descriptive analyses summarized the distribution of key sociodemographic factors and the prevalence of household access to improved water sources, improved toilets, and basic sanitation. Before modelling, we created a new standardized survey- weight to ensure that each country contributed proportionately to the pooled regional estimates. Specifically, the original DHS weights (v005) were first normalized, then rescaled within each country by dividing by the country’s total weight and multiplying by the average total weight across all countries. This was necessary because DHS survey weights are country‑specific and therefore not directly comparable in multi‑country pooled analyses. The standardized weight adjusted for differences in sample size and prevented countries with larger DHS samples from disproportionately influencing the pooled estimates.

To examine determinants of household access to improved water, improved toilet facilities, and basic sanitation, we implemented two complementary modelling approaches: We first ran survey‑weighted Poisson regression models with robust variance estimation to obtain prevalence ratios (IRRs) for each outcome. These models incorporated: The standardized survey weights, adjustment for clustering and stratification using *svyset*. All independent variables entered simultaneously using a forced‑entry approach. In the fixed‑effects models, country was included as a categorical predictor, producing country‑specific slope estimates relative to the reference country (Angola). These models addressed observed, measurable cross‑country variation but could not fully account for unobserved country‑level heterogeneity.

To further control for unobserved heterogeneity across countries, we fitted random‑intercept multilevel or Random effect Poisson regression models, with households nested within countries. This approach allowed country intercepts to vary, thereby capturing contextual effects that were not measured at the household level (e.g., differences in national infrastructure, policy, governance, or sanitation programs). In this approach we quantified the between‑country variance and intraclass correlation coefficients (ICC) for each outcome. Because our outcomes are common (prevalence >10%), odds ratios from logistic regression would not approximate risk/prevalence ratios and can overstate associations, in part due to the non‑collapsibility of the odds ratio. We therefore estimated prevalence ratios (PRs) using Poisson regression with a log link and robust (sandwich) variance (“modified Poisson”), which yields consistent PR estimates and valid confidence intervals for binary outcomes, while avoiding the convergence problems common in log‑binomial models. All models incorporated [18,19] the complex survey design (stratification, clustering, and weights) using design‑based variance estimation

The authors used Strengthening the Reporting of Observational studies in Epidemiology (STROBE) reporting guidelines to guide the reporting of this study

## Results

### Description of sample

Table 1 displays weighted samples and survey years for each country included in the study. The majority (42.1%) of the data was collected from 2019 to 2024. Nigeria and Kenya contained 9.0% and 6.9% of households, respectively, with the remaining countries containing less than 6% of households in the sample. However, the Democratic Republic of Congo had less than 1% of households in the sample.

Table 2 presents the characteristics of the study population. A total of 465,208 households were included in this study for analysis. The majority (60.8%) of respondents were rural residents. Less than one-third (29.4%) of respondents had no formal education, and a few (5.5%) had higher education. About 34.6% of respondents attained secondary level education. More than 7 in 10 (70.9%) respondents were from male-headed households.

**Table 2:** Distribution of weighted samples of demographic and health survey data from 30 countries.

Variables	Frequency (N = 465,208)	Percentage (%)
**Place of residence**		
Urban	182,222	39.17
Rural	282,986	60.83
**Educational Level**		
No education	136,736	29.39
Primary	141,812	30.48
Secondary	160,895	34.59
Higher	25,765	5.54
**Sex of household head**		
Male	329,874	70.91
Female	135,334	29.09
**Wealth index**		
Poorest	92,703	19.93
Poorer	88,784	19.08
Middle	92,974	19.99
Richer	94,384	20.29
Richest	96,363	20.71
**Year of interview**		
2014-2016	132,546	26.60
2017-2019	146,013	31.40
>2019	195,649	42.10

### Prevalence of household access to and use of improved water sources, toilet facilities, and basic sanitation in 30 SSA countries

Table 3 presents the prevalence analysis of access to and use of improved water sources, toilet facilities, and basic sanitation facilities. In the 30 countries in SSA analyzed, 76.3% of households have access to and use improved water sources. Additionally, 56.2% of households have access to improved toilet facilities, with 35.4% having access to basic sanitation facilities.

**Table 3:** Prevalence of household access to and use of improved water sources, toilet facilities, and basic sanitation in 30 SSA countries, and their distribution.

Variable	Improved Water N=346,938 N (%)	P-Value	Improved Toilet N=255,569 N (%)	P-Value	Basic Sanitation (Unshared improved toilet) N=128,691 N (%)	P-Value
**Overall Prevalence**	346,938 (76.3)		255,569 (56.2)		128,691 (35.4)	
**Sex**		<0.001		<0.001		<0.001
Male	240785 (74.53)		176180 (55)		86,119 (33.7)	
Female	106153 (80.73)		79389 (60)		42572 (39.46)	
**Residence**		<0.001		<0.001		<0.001
Urban	164540 (92.61)		138494 (77.96)		65854 (40)	
Rural	182398 (65.88)		117075 (42.29)		62837 (31.6)	
**Wealth Index**		<0.001		<0.001		<0.001
Poorest	47981 (52.72)		17998 (19.78)		17445 (35.01)	
Poorer	57049 (65.65)		33222 (38.23)		21737 (34.61)	
Middle	69955 (77.02)		51087 (56.25)		27105 (36.28)	
Richer	81130 (88.04)		68739 (75)		34682 (41.46)	
Richest	90823 (96.98)		84523 (90)		27722 (29.97)	
**Education**		<0.001				<0.001
No education	89370 (66.18)		51997 (38.52)		32207 (36.75)	
Primary	100649 (72.44)		72792 (52.39)		38895 (34.5)	
Secondary	133433 (85.57)		108.754 (69.74)		51071 (36.73)	
Higher	23486 (95.43)		22026 (89.5)		6518 (27.12)	
**Country**		<0.001				<0.001
Angola	9476 (66.73)		8492 (59.8)		3237 (32.85)	
Benin	11050 (70.94)		5246 (33.68)		4205 (59.18)	
Burkina Faso	14702 (84.33)		11295 (64.79)		4902 (41.11)	
Burundi	14385 (83.92)		9976 (58.2)		2734 (16.23)	
Cameroon	10640 (77.2)		8396 (60.92)		3310 (24.92)	
Chad	9892 (56.41)		2454 (14.05)		2170 (39.57)	
Cote d’ivoire	12202 (84.01)		8733 (60.13)		5277 (45.51)	
DR Congo	1185 (28.66)		1628 (39.37)		1691 (48.73)	
Gabon	8536 (79.76)		5857 (54.73)		3540 (33.53)	
Gambia	10868 (94.83)		7089 (61.85)		2816 (24.92)	
Ghana	12567 (85.33)		8132 (55.22)		6156 (61.02)	
Guinea	8652 (80.66)		5713 (53.26)		4412 (47.07)	
Kenya	23652 (75.98)		20629 (66.27)		10782 (38.55)	
Lesotho	5314 (89.46)		3770 (63.47)		1617 (34.15)	
Liberia	6394 (81.11)		3070 (38.94)		2721 (58.93)	
Madagascar	9221 (49.53)		6133 (32.94)		7761 (63.63)	
Malawi	21354 (87.79)		20688 (85.05)		7867 (34.03)	
Mali	7712 (74.01)		5880 (56.43)		3725 (40.78)	
Mauritania	12720 (82.84)		9653 (62.87)		1803 (16.66)	
Mozambique	9336 (72.07)		4153 (32.06)		1495 (14.18)	
Nigeria	29266 (70.89)		21895 (53.03)		9965 (32.7)	
Rwanda	11647 (81.01)		10550 (73.38)		2243 (16)	
Senegal	14130 (87.27)		11811 (72.95)		2993 (20.19)	
Sierra Leone	10097 (65.73)		8444 (54.97)		8756 (69.05)	
South Africa	7680 (93.25)		5811 (70.56)		1271 (15.9)	
Tanzania	11272 (76.38)		11437 (77.5)		3037 (22.46)	
Togo	3391 (60.43)		1581 (28.16)		1256 (60.65)	
Uganda	14275 (79.96)		6388 (35.78)		6519 (39.49)	
Zambia	9444 (70.79)		7145 (53.56)		4150 (34.31)	
Zimbabwe	15878 (83.74)		13520 (71.3)		6280 (41.16)	

Some variations were observed in access to improved water based on wealth quintile and place of residence. Wealthier households (richer and richest) had nearly universal access, with 98.8% and 97.0%, respectively. In contrast, the poorest households had only 52.7.0% access. Urban households had significantly higher access (92.6%) compared to rural households (65.9%). Significant differences were also noted by country, with Gambia, and Senegal showing the highest access, while DR Congo and Chad had the lowest (Table 3).

Access to improved toilet facilities was significantly associated with wealth quintile, place of residence, and education level. Wealthier households (richer and richest) had 90% access to, and use compared to poorest households (19.8%) Urban households showed higher (78%) percentage access to and use (89.0%) compared to rural households (42.3%) Household heads with higher education indicated a higher access rate (89.5%) than those without education (38.5%) depicting a wider need gap. Significant country differences were also observed, with Rwanda, Senegal (81.6%), Tanzania (77.52%), Zimbabwe (71.3.3%), and Rwanda (73.4%) showing high access rates (Table 3).

Significant disparities in access to basic sanitation were observed between household wealth quintile and place of residence. Urban households indicated 40% access, and 41.5% for richer households. There were also notable country differences, with high access rates in Senegal (69.1%) and, (63.6%), while countries like Mauritania (14.18) and South Africa (15.9%) showed lower access rates. (Table 3).

### Factors associated with access to and use of improved water sources, improved toilets, and basic sanitation facilities

#### Factors associated with access to and use of improved water sources.

**Fixed effect model:** In Table 4, Urban residence was associated with higher prevalence (*IRR* = 0.60, 95% CI [0.37, 0.96]) of access compared with urban residence. Primary education (vs. none) was associated with higher prevalence of access (*IRR* = 1.20, 95% CI [1.09, 1.32]) compared to secondary education. For countries (relative to Angola), significantly higher prevalence of improved water access was observed in Malawi (*IRR* = 1.50, 95% CI [1.25, 1.80]) and Senegal (*IRR* = 1.42, 95% CI [1.28, 1.57]).

**Table 4:** Factors associated with access to and use of improved water sources, improved toilets, and basic sanitation facilities.

Variables	Access to and use of improved water		Access to and use of improved toilet		Access to and use of basic sanitation	
	Fixed Effect	Random Effect	Fixed Effect	Random Effect	Fixed Effect	Random Effect
	IRR (95% CI)	IRR (95% CI)	IRR (95% CI)	IRR (95% CI)	IRR (95% CI)	IRR (95% CI)
**Household wealth**						
Poorest	Reference		Reference		Reference	
Poorer	1.44(0.90, 2.29)	1.23 (1.22, 1.25)	2.56(2.15, 3.05)	1.93 (1.89, 1.96)	1.02 (1.01, 1.04)	1.02 (1.01, 1.04)
Middle	1.23(0.92, 1.66)	1.41 (1.39, 1.43)	6.36(4.20, 9.61)	2.74 (2.69, 2.79)	1.04 (1.03, 1.06)	1.04 (1.03, 1.06)
Richer	1.02(0.70, 1.49)	1.55 (1.53, 1.57)	7.11(4.87, 10.38)	3.44 (3.37, 3.50)	1.03 (1.01, 1.05)	1.03 (1.01, 1.05)
Richest	1.20(0.90, 1.65)	1.62 (1.60, 1.65)	7.52(5.28, 10.72)	3.96 (3.88, 4.03)	1.35 (1.33, 1.38)	1.35 (1.33, 1.38)
**Sex of Household head**						
Male	Reference		Reference			
Female	1.05(0.92, 1.20)	1.04 (1.03, 1.04)	1.10(1.00, 1.21)	1.03 (1.02, 1.04)	0.92 (0.91, 0.93)	0.92 (0.91, 0.93)
**Type of residence**						
Urban	Reference		Reference			
Rural	0.60(0.37, 0.96)	0.87 (0.86, 0.88)	1.02(0.95, 1.08)	0.88 (0.87, 0.89)	1.34 (1.32, 1.37)	1.34 (1.33, 1.36)
**Educational level**						
No education	Reference		Reference			
Primary	1.20(1.09, 1.32)	1.02 (1.01, 1.03)	0.86(0.62, 1.19)	1.08 (1.06, 1.09)	0.99 (0.98, 1.00)	0.99 (0.97, 1.00)
Secondary	0.99(0.81, 1.20)	1.06 (1.05, 1.07)	1.21(1.11, 1.31)	1.11 (1.09, 1.12)	0.99 (0.97, 1.00)	0.99 (0.97, 1.00)
Higher	1.09(1.00, 1.18)	1.04 (1.03, 1.06)	1.14(1.07, 1.20)	1.12 (1.11, 1.14)	1.15 (1.13, 1.17)	1.15 (1.13, 1.17)
**Country**						
Angola	Reference	0.91 (0.89, 0.93)	Reference	1.17 (1.14, 1.19)		1.27 (1.24, 1.30)
Benin	1.12(1.01, 1.24)	0.93 (0.91, 0.95)	0.51(0.47, 0.55)	0.63 (0.61, 0.64)	0.52 (0.49, 0.55)	0.66 (0.64, 0.69)
Burkina Faso	1.43(1.23, 1.66)	1.13 (1.11, 1.15)	0.99(0.94, 1.03)	1.21 (1.19, 1.23)	0.77 (0.73, 0.81)	0.98 (0.96, 1.00)
Burundi	1.53(1.23, 1.91)	1.17 (1.15, 1.19)	0.86(0.81, 0.91)	1.04 (1.02, 1.06)	1.02 (0.98, 1.07)	1.30 (1.28, 1.32)
Cameroon	1.15(1.05, 1.26)	0.99 (0.97, 1.01)	0.89(0.84, 0.94)	1.07 (1.05, 1.10)	1.04 (0.99, 1.08)	1.32 (1.29, 1.34)
Chad	1.03(0.87,1.22)	0.80 (0.79, 0.82)	0.26(0.22, 0.29)	0.31 (0.30, 0.32)	0.70 (0.66, 0.74)	0.89 (0.86, 0.92)
Cote d’Ivoire	1.27(1.19, 1.34)	1.10 (1.08, 1.12)	1.00(0.96, 1.04)	1.19 (1.16, 1.21)	0.78 (0.73, 0.83)	0.99 (0.96, 1.01)
DR Congo	0.78(0.54, 1.14)	0.63 (0.60, 0.66)	0.66(0.46, 0.92)	0.80 (0.76, 0.84)	0.56 (0.46, 0.68)	0.71 (0.68, 0.75)
Gabon	1.18(1.10, 1.27)	1.13 (1.11, 1.16)	1.06(0.98, 1.13)	1.28 (1.25, 1.31)	1.02 (0.97, 1.08)	1.30 (1.27, 1.33)
Gambia	1.31(1.23, 139)	1.18 (1.16, 1.20)	1.02(0.96, 1.07)	1.21 (1.19, 1.24)	1.08 (1.03, 1.13)	1.37 (1.34, 1.40)
Ghana	1.35(1.19, 1.53)	1.13 (1.11, 1.15)	0.90(0.82, 0.99)	1.15 (1.13, 1.17)	0.51 (0.47, 0.56)	0.65 (0.63, 0.67)
Guinea	1.36(1.21, 1.54)	1.09 (1.07, 1.12)	0.85(0.81, 0.89)	1.03 (1.00, 1.05)	0.67 (0.63, 0.72)	0.86 (0.83, 0.88)
Kenya	1.23(1.06, 1.43)	1.02 (1.01, 1.03)	1.01(0.96, 1.04)	1.22 (1.21, 1.24)	0.79 (0.76, 0.84)	1.01 (1.00, 1.03)
Lesotho	1.43(1.20, 1.71)	1.15 (1.12, 1.18)	0.91(0.83, 0.99)	1.16 (1.13, 1.20)	0.82 (0.77, 0.88)	1.05 (1.01, 1.08)
Liberia	1.25(1.16, 1.36)	1.09 (1.06, 1.12)	0.73(0.67, 0.79)	0.89 (0.86, 0.92)	0.61 (0.55, 0.68)	0.78 (0.75, 0.81)
Madagascar	0.86(0.70, 1.07)	0.67 (0.65, 0.68)	0.53(0.49, 0.57)	0.65 (0.64, 0.67)	0.45 (0.42, 0.48)	0.57 (0.55, 0.58)
Malawi	1.50(1.25, 1.80)	1.21 (1.19, 1.22)	1.39(1.27, 1.52)	1.61 (1.58, 1.63)	0.79 (0.75, 0.82)	1.00 (0.98, 1.02)
Mali	1.28(1.07, 1.53)	0.97 (0.95, 1.00)	0.91(0.85, 0.96)	1.12 (1.09, 1.15)	0.73 (0.69, 0.77)	0.93 (0.91, 0.95)
Mauritania	1.20(1.11, 1.31)	1.04 (1.02, 1.06)	0.95(0.91, 0.09)	1.11 (1.09, 1.13)	1.15 (1.10, 1.20)	1.46 (1.43, 1.49)
Mozambique	1.04(0.90, 1.19)	0.86 (0.84, 0.88)	0.48(0.44, 0.51)	0.56 (0.55, 0.58)	1.13 (1.09, 1.18)	1.44 (1.41, 1.47)
Nigeria	1.18(1.06, 1.32)	0.97 (0.96, 0.98)	0.81(0.76, 0.86)	1.00 (0.99, 1.01)	0.84 (0.80, 0.88)	1.07 (1.06, 1.09)
Rwanda	1.37(1.13, 1.66)	1.10 (1.08, 1.12)	1.18(1.09, 1.26)	1.38 (1.36, 1.41)	1.03 (0.99, 1.07)	1.31 (1.28, 1.33)
Senegal	1.42(1.28, 1.57)	1.18 (1.17, 1.20)	1.20(1.14, 1.25)	1.45 (1.43, 1.48)	1.07 (1.03, 1.12)	1.36 (1.34, 1.39)
Sierra Leone	1.11(0.96, 1.29)	0.90 (0.88, 0.92)	0.84(0.78, 0.90)	1.04 (1.02, 1.06)	0.43 (0.40, 0.46)	0.54 (0.53, 0.56)
South Africa	1.35(1.19, 1.53)	1.17 (1.15, 1.20)	1.03(0.92, 1.14)	1.31 (1.27, 1.34)	1.18 (1.12, 1.23)	1.49 (1.46, 1.53)
Tanzania	1.17(1.02, 1.33)	0.97 (0.95, 0.99)	1.16(1.08, 1.24)	1.35 (1.32, 1.37)	0.94 (0.90, 0.99)	1.20 (1.18, 1.22)
Togo	0.99(0.84, 1.17)	0.82 (0.79, 0.85)	0.53(0.46, 0.60)	0.63 (0.60, 0.66)	0.53 (0.47, 0.60)	0.68 (0.64, 0.73)
Uganda	1.31(1.12, 1.53)	1.07 (1.06, 1.09)	0.63(0.58, 0.68)	0.74 (0.72, 0.75)	0.77 (0.73, 0.81)	0.98 (0.96, 1.00)
Zambia	1.14(1.00, 1.29)	0.96 (0.94, 0.98)	0.85(0.80, 0.89)	1.01 (0.98, 1.03)	0.81 (0.76, 0.86)	1.03 (1.01, 1.05)
Zimbabwe	1.25(1.14, 1.38)	1.02 (1.00, 1.04)	0.90(0.79, 1.01)	1.18 (1.16, 1.20)	0.78 (0.73, 0.83)	0.99 (0.97, 1.01)
Variance (Country RE)		0.026		0.114		0.081
ICC (Country)		0.008		0.034		0.024

IRR = Incident Rate Ratio, CI = Confidence Interval.

**Random effect:** Accounting for clustering by country, wealth showed clear positive gradients (e.g., richest vs. poorest: *IRR* = 1.62, 95% CI [1.60, 1.65]), Education showed positive associations (e.g., secondary vs. none: *IRR* = 1.06, 95% CI [1.05, 1.07]; higher: *IRR* = 1.04, 95% CI [1.03, 1.06]), and female‑headed households had slightly higher prevalence (*IRR* = 1.04, 95% CI [1.03, 1.04]). Country effects indicated deviations from the average country: for example, Gambia had higher‑than‑average prevalence (*IRR* = 1.18, 95% CI [1.16, 1.20]) than Angola (*IRR* = 0.91, 95% CI [0.89, 0.93]).

Between‑country variance and ICC: Country‑level variance was 0.026 with ICC = 0.008, indicating that about 0.8% of residual variation in improved water access prevalence was attributable to between‑country differences after adjusting for possible confounders.

#### Factors associated with access to and use of improved toilet facilities.

**Fixed effect:** Wealth was the strongest determinant of prevalence. Prevalence increased progressively across wealth groups, poorer (*IRR* = 2.56, 95% CI [2.15, 3.05]), middle (*IRR* = 6.36, 95% CI [4.20, 9.61]), richer (*IRR* = 7.11, 95% CI [4.87, 10.38]), and richest (*IRR* = 7.52, 95% CI [5.28, 10.72]). Secondary (*IRR* = 1.21, 95% CI [1.11, 1.31]) and Higher education (*IRR* = 1.14, 95% CI [1.07, 1.20]) were associated with higher prevalence than primary education which was not significant (*IRR* = 0.86, 95% CI [0.62, 1.19]). Female‑headed households showed marginally higher prevalence (*IRR* = 1.10, 95% CI [1.00, 1.21]) than male headed households. Country differences (relative to Angola) ranged widely, from markedly lower prevalence in Chad (*IRR* = 0.26, 95% CI [0.22, 0.29]) to higher prevalence in Malawi (*IRR* = 1.39, 95% CI [1.27, 1.52]), Senegal (*IRR* = 1.20, 95% CI [1.14, 1.25]), and Tanzania (*IRR* = 1.16, 95% CI [1.08, 1.24]).

**Random effect:** Wealth remained strongly associated with prevalence but at reduced magnitudes. poorer (*IRR* = 1.93, 95% CI [1.89, 1.96]), middle (*IRR* = 2.74, 95% CI [2.69, 2.79]), richer (*IRR* = 3.44, 95% CI [3.37, 3.50]), and richest (*IRR* = 3.96, 95% CI [3.88, 4.03]). Rural residence was associated with lower prevalence (*IRR* = 0.88, 95% CI [0.87, 0.89]). Education was positively associated at all levels (e.g., higher: *IRR* = 1.12, 95% CI [1.11, 1.14]). Female‑headed households had slightly higher prevalence (*IRR* = 1.03, 95% CI [1.02, 1.04]). Country deviations from the cross‑country mean were large; for example, Senegal had higher‑than‑average prevalence (*IRR* = 1.45, 95% CI [1.43, 1.48]) while Chad had lower‑than‑average prevalence (*IRR* = 0.31, 95% CI [0.30, 0.32]).

Between‑country variance and ICC: Country‑level variance was 0.114 with ICC = 0.034, meaning 3.4% of residual variation in improved toilet prevalence was attributable to between‑country differences.

#### Factors associated with access to and use of basic sanitation facilities (unshared improved toilet facilities).

**Fixed effect:** Wealth was associated with increased prevalence in a graded pattern, poorer (*IRR* = 1.02, 95% CI [1.01, 1.04]), middle (*IRR* = 1.04, 95% CI [1.03, 1.06]), richer (*IRR* = 1.03, 95% CI [1.01, 1.05]), and richest (*IRR* = 1.35, 95% CI [1.33, 1.38]). Rural households had higher prevalence than urban households (*IRR* = 1.34, 95% CI [1.32, 1.37]). Female‑headed households had lower prevalence (*IRR* = 0.92, 95% CI [0.91, 0.93]). Education was significant only at the highest level (higher vs. none: *IRR* = 1.15, 95% CI [1.13, 1.17]). Relative to Angola, South Africa (*IRR* = 1.18, 95% CI [1.12, 1.23]) and Mozambique (*IRR* = 1.13, 95% CI [1.09, 1.18]) had higher prevalence.

**Random effect:** Wealth remained positively associated with basic sanitation prevalence. Rural residence was associated with higher prevalence (*IRR* = 1.34, 95% CI [1.33, 1.36]). Higher education remained significant (*IRR* = 1.15, 95% CI [1.13, 1.17]). Countries showed both positive and negative deviations from the mean. For example, while, Mauritania had higher‑than‑average prevalence (*IRR* = 1.46, 95% CI [1.43, 1.49]), Sierra Leone had lower‑than‑average prevalence (*IRR* = 0.54, 95% CI [0.53, 0.56]). Between‑country variance and ICC: Country‑level variance was 0.081 with ICC = 0.024, meaning 2.4% of residual variation in improved toilet prevalence was attributable to between‑country differences.

## Discussion

This paper examined the proportion of households with access to and use of improved water sources, improved toilet and basic sanitation facilities. Additionally, it assessed factors associated with the phenomenon in Sub-Saharan Africa (SSA). The findings indicate that access to and use of improved water, improved toilets, and basic sanitation facilities in SSA remains a significant challenge. About 23.7% of households in SSA still lack access to safe drinking water. Though the observed level of access is a valuable achievement in the context of global development goals, nearly a quarter of the population continues to experience substantial deficits. Access to improved water is critical for reducing the burden of diseases such as cholera and diarrhea, which disproportionately affect vulnerable populations in low-income regions [4,6,20]. Studies have indicated that lack of access to safe water can contribute to the onset of waterborne diseases [3,17].

In contrast, access to basic sanitation facilities, defined as improved toilet facilities not shared with other households, shows a more worrying situation, as many households still lack access to improved toilet facilities. The findings revealed that many households continue to rely on inadequate or unsafe sanitation sources, such as pit latrines or open defecation, which can potentially expose individuals and communities to harmful pathogens and contribute to the spread of diseases [2,3,17]. This disparity underscores the challenges of achieving universal safe sanitation in SSA, where persistent infrastructural, economic, and social barriers remain [1,3,21].

Across the outcomes (improved water, improved toilet and basic sanitation), the fixed- effect and multilevel (random- effect) results showed a generally consistent pattern. The multilevel model refined varied from the survey-weighted estimates, suggesting that the socioeconomic gradients driving coverage are robust to model choice. The most stable determinants of higher prevalence across models were household wealth and educational attainment. This aligns with evidence in both national and cross-country studies showing that wealthier and educated households are more likely to access improved water and sanitation services [22–24]. Conversely, poorest households in low-income populations appeared to remain largely excluded from formal water supply and sanitation systems [1,2,4,17].

The influence of wealth on prevalence in both models align with patterns of coverage in existing literature [1,17,21] and the indication that household wealth improves consistent and secure access and use likely due to the economic capacity to afford and maintain such infrastructure.

Similarly, the findings that household educational level determine access, and use is supported by previous studies which indicate that individuals with higher education are more likely to be well informed about sanitation practices and their benefits. Thus, individuals with higher education may be better equipped financially to invest in sanitation facilities than poorer households [2,10,12,25,26].

Residential areas differed by the outcomes of this study but were stable across the models and consistent with existing research. Rural residence was associated with lower access and use of improved water sources but showed higher prevalence for basic sanitation. Though the finding appears counterintuitive, it may reflect a lower likelihood of shared toilet use in rural areas. These findings across the models indicate robust residence effects that persist after accounting for community clustering. Similar contextual interpretation issues have been noted in the literature where rural-urban differences may reflect service typology rather than pure access advantages [22,24]. This disparity could be explained by the more infrastructure in urban areas due to increased population densities and demand [2,4,17], and the challenges with maintenance and sustainability of the limited sanitation investments [21,22].

Wealth, education, and sex of household head showed relatively modest but consistent associations with water and sanitation outcomes. Across models, female-headed households reported slightly higher prevalence of improved toilet access but lower prevalence of basic sanitation. Other studies similarly show that female-headed households often have higher odds of accessing improved water or sanitation compared to male-headed households [3,7]. These patterns likely reflect contextual differences in the types of sanitation facilities accessible to male-versus female-headed households, particularly relating to whether toilets are shared or private. This is supported by evidence indicating that this pattern is likely driven by concerns for privacy, safety and dignity, which are especially important for women and girls when toilets are shared or located outside the household [1,4,27]. Country level effects differed in interpretation across models but were internally coherent, reflecting distinct national coverage patterns. In the fixed-effect model, countries were compared with Angola, producing clear contrast in prevalence across settings. In the multilevel model, countries were compared with the average country, revealing how far individual countries deviated from the regional means after accounting for household-level characteristics. Though the reference points differed, both models consistently identified certain countries such as Malawi, Senegal and South Africa as having higher prevalence for at least one outcome, whereas countries like Chad, Sierra Leone, and DR Congo showed lower prevalence. This reflects broader SSA monitoring data showing large cross-country disparities in access to improved water and sanitation [28,29]. Multilevel and spatial analysis across SSA also identify hotspots of unimproved sanitation that correspond to specific national contexts, with Malawi exhibiting low prevalence of unimproved sanitation and countries like Chad exhibiting some of the highest levels, reinforcing findings from this study [29].

The random-effects results further demonstrated that only a small proportion of the remaining variability was attributable to differences in between-country, as reflected by low ICC values (0.8% for improved water, 3.4% for improved toilet, and 2.4% for basic sanitation). This suggests that most of the variations in coverage could be explained by household-level characteristics rather than country-level clustering though improved toilet access showed somewhat greater contextual heterogeneity.

Overall, both models provide a coherent narrative that, socio-economic status, education and place of residence are the primary drivers of household-level differences in access to and use of improved water and sanitation services across the 30 SSA countries, while country-level differences contribute additional but comparatively smaller contextual variations.

### Policy implications

The current study highlights a significant lack of access to and use of improved water sources, improved toilet and basic sanitation facilities among households in the sample, a challenge that continues to affect the SSA region. The implications of these findings are significant for public health planning and the achievement of the SDGs. There is an urgent need for increased investment in water and sanitation infrastructure, particularly in countries where access remains critically low [5,7,30]. This may include upgrading infrastructure for safe water and sanitation, raising public awareness and education, and empowering local communities to establish and maintain effective water and sanitation systems [7,30].

### Strengths and limitations

A significant strength of this study is the use of pooled nationally representative DHS datasets covering 30 SSA countries, which enhances statistical power and enabled cross-country comparability, making findings generalizable to households across SSA. The pooled approach also allows for regional-level inferences and provides insight relevant for monitoring progress toward global targets such as the SDGs. In addition, the appropriate handling of the complex survey design through the application of survey weights and clustering (svyset) strengthens the robustness and validity of the estimates.

However, the use of pooled datasets may also mask important contextual differences between countries. While pooling improves regional inference, it may obscure substantial between-country heterogeneity in infrastructure development, governance, and socioeconomic gradients. Consequently, regional averages may not fully reflect country-specific realities, and exposure outcome relationships may vary across national contexts. In smaller countries or settings with unique service delivery patterns, pooling may therefore conceal important or contrasting associations.

Despite these limitations, the findings provide important insights into the persistent gaps in access to and use of improved water and sanitation facilities across SSA. The results underscore the continuing disparities in service coverage and highlight the need for targeted interventions to address structural inequalities in access. Of critical importance is that safe water and basic sanitation remain fundamental requirements for human health, well-being, and sustainable development.

## Conclusion

Access to and use of improved water and sanitation facilities in SSA continues to pose a significant challenge, as many households still lack access to improved water sources and sanitation services. Households still grapple with lack of access mainly due to socio-economic, educational and geographical factors. This urgently calls for water and sanitation initiatives that seek to provide sustainable and equitable solutions. The pooled findings indicate that regional averages are useful for benchmarking progress toward SGD 6, these can mask substantial within-country disparities that require targeted national responses. These findings underscore the need for context-specific interventions that address household-level factors affecting access to and use of improved water and sanitation facilities in SSA.
